# Genetic variability of *Haemonchus contortus* isolates in small ruminants from slaughterhouses in Bangladesh

**DOI:** 10.1007/s00436-023-08000-4

**Published:** 2023-10-19

**Authors:** Muhammad Abdul Mannan, Sharmin Chowdhury, Mohammad Alamgir Hossain, Md Hazzaz Bin Kabir

**Affiliations:** 1 Department of Microbiology and Parasitology, Sher-e-Bangla Agricultural University, Sher-e-Bangla Nagar, Dhaka, 1207 Bangladesh; 2 Department of Pathology and Parasitology, Faculty of Veterinary Medicine, Chattogram Veterinary and Animal Sciences University, Khulshi, Chittagong, 4225 Bangladesh; 3 Laboratory of Sustainable Animal Environment, Graduate School of Agricultural Science, Tohoku University, 232-3 Yomogida, Naruko-onsen, Osaki, Tohoku, Miyagi 989-6711 Japan

**Keywords:** *Haemonchus contortus*, Genetic variation, ITS-2 gene, *nad*4 gene, Small ruminant, Slaughterhouse

## Abstract

*Haemonchus contortus* is a blood-sucking gastrointestinal nematode that infects all ruminants and causes significant economic losses in production. Characterizing the genetic variability of *H. contortus* populations is crucial for understanding patterns of disease transmission and developing effective control strategies against haemonchosis. This study aimed to identify the genetic variability of *H. contortus* isolates in small ruminants from slaughterhouses in Bangladesh. During January to December 2015, 400 abomasa samples were collected and 186 were found to be positive for *Haemonchus*. A 321-bp fragment of the second internal transcribed spacer (ITS-2) of nuclear ribosomal DNA and an 800-bp fragment of the mitochondrial nicotinamide dehydrogenase subunit-4 gene (*nad*4) were amplified using polymerase chain reaction (PCR) and directly sequenced. The results showed 10 genotypes (ITS-2) and 45 haplotypes (*nad*4) among the 186 worms. The sequences were 98.5 to 100% identical to reference sequences from the GenBank database. ITS-2 sequence analysis revealed four nucleotide substitutions at positions 30, 41, 42, and 216. There was one transition (C/T) at position 42 and three transversions (C/A at position 30, G/C at position 41, and T/A at position 216). The *nad*4 gene sequences showed 15 substitutions, all of which were transitions. The pairwise distance of ITS-2 between *H. contortus* populations ranged from 0.005 to 1.477. The nucleotide diversity (*μ*) among the populations was 0.009524 using ITS-2 and 0.00394 using *nad*4. This study indicated low genetic deviation among *H. contortus* populations in Bangladesh.

## Introduction

*Haemonchus contortus* is a blood-sucking gastrointestinal nematode that infects ruminants and is one of the major pathogens affecting small ruminants worldwide (O’Connor et al. 2006). The infection, known as haemonchosis, results in significant economic losses in meat, milk, and leather production, and is characterized by clinical signs such as anemia, edema, and death due to blood loss (Easwaran et al. 2009). This parasite is primarily responsible for causing illness and death in infected animals during the summer months in warm and humid climates (Gasser and Newton 2000).

The parasite *H. contortus* has a high degree of genetic variability (Anderson et al. 1998). Studies on the population genetics of *H. contortus* in the USA have shown high within-population variation and low genetic differentiation within continuous geographical regions (Blouin et al. 1995 and Anderson et al. 1998). Similar studies have been conducted in various regions around the world, including Australia, Brazil, Europe, and the surrounding countries of Bangladesh (India, Pakistan, Thailand, Malaysia, and China), and have found genetic variation and relatively low host specificity for *H. contortus* (Gasser et al. 1998; Troell et al. 2006; Hunt et al. 2008; Cerutti et al. 2010; Brasil et al. 2012; Gharamah et al. 2012; Yin et al. 2013). Additionally, this parasite has the ability to develop resistance to anthelmintics and has the capacity to survive due to its biological and ecological plasticity (Troell et al. 2006).

The Second Internal Transcribed Spacer (ITS-2) of nuclear ribosomal DNA (rDNA) has been developed as a reliable genetic marker for strongylid species identification (Bott et al. 2009; Gharamah et al. 2012) due to its high inter-specific sequence divergence and intra-specific sequence homogeneity (Heise et al. 1999). The ITS-2 has been frequently used in species identification within the genus *Haemonchus* (Gasser et al. 1998; Heise et al. 1999). Moreover, the intra-specific differentiation of ITS-2 within *H. contortus* ranges from 0 to 5.2% (Stevenson et al. 1995; Zarlenga et al. 1998; Heise et al. 1999).

It is generally easy to distinguish between closely related individuals because mtDNA undergoes more substitutions than nuclear DNA (Blouin 2002). As the variation in cytochrome oxidases is primarily restricted to silent sites due to strong amino acid conservation, McDonnell et al. (2000) discovered that protein coding loci, such as the *nad*4 gene in the mitochondrial nicotine amide dehydrogenase group, are more effective for prospecting than CO genes. Therefore, the *nad*4 gene has been extensively utilized in prior studies on the *H. contortus* population (Troell et al. 2006; Cerutti et al. 2010). In contrast, information on genetic variation of this worm is limited in Bangladesh which is crucial for studying genetic characterization, molecular epidemiology and to develop the effective control strategies. So, the present study was conducted genetic variation within and among the populations of *H. contortus* isolated in small ruminant from slaughterhouse in Bangladesh amplifying the ITS-2 of nuclear rDNA and the *nad*4 genes.

## Materials and methods

### Ethics statement

This study was approved by the ethical committee of Chattogram Veterinary and Animal Sciences University authority and Chattogram City Corporation, Bangladesh.

### Parasite material

The present study was carried out on slaughtered goats and sheep reared in various geographic regions (coastal, plain, and hilly) of Bangladesh but slaughtered in local abattoirs of Chattogram metropolitan area (geographical coordinates: 22°21′94″ North, 91°48′12″ East). Abomasal samples were collected once in a week from the selected abattoirs situated at Jautala, Firingybazar, Pahartali, Colonethat, and Halishahar. A total of 400 abomasal samples were collected during the period of January to December, 2015. The both ends of the each abomasum was ligated and transferred to plastic zipper bags to avoid contamination and then transported to the Pathology and Parasitology departmental laboratory at Chittagong Veterinary and Animal Sciences University (CVASU) in Bangladesh. The adult worms were collected from the abomasa according to the protocol described by Hansen and Perry (1994) and Iqbal et al. (1993) with some modifications. The collected parasites were washed by normal saline and placed in sterile petridishes. The collected worms were examined by naked eye and then under microscope according to the procedure given by Soulsby (1982) for species identification. The identified *Haemonchus* species were pooled by grouping of all worms found per abomasum.

### Isolation of genomic DNA

Total genomic DNA was extracted using G-spin^TM^ Total DNA Extraction Kit (REF-17045, iNtRON Biotechnology, Korea; web site: http://www.intronbio.com) according to the manufacturer’s instruction. Extracted DNA was stored at −20°C temperature until use.

### PCR amplification and sequencing

A single-step PCR was conducted to amplify the 321 bp of ITS-2 of nuclear rDNA using the forward primer NC1-F (5′-ACGTCTGGTTCAGGGTTGTT-3′) and the reverse primer NC1-R (5′-TTA GTT TCT TTT CCT CCG CT-3′) as shown in Table 1 (Akkari et al. 2013). The 800 bp of the *nad*4 gene was also amplified using Primer1-F (GGATTTGGTCAGCAAATTGAA) and Primer2-R (GCCTGCAAATGAATTAACA) (Yin et al. 2013). Each PCR was performed in a 50-μl reaction containing a master mix of 25 μl (DNA polymerase, buffer, 0.4 mM of each dATP, dCTP, dGTP, and dTTP, 4 mM of MgCl2; iNtRON Biotechnology; web site URL: http://www.intronbio.com), 5 μl of DNA template (above 30 ng/μl), 2.5 μl of forward primer (10 pmol/μl), 2.5 μl of reverse primer (10 pmol/μl), and 15 μl of distilled water. The PCR was carried out in a Thermal Cycler (2720 Thermal Cycler; Applied Biosystems). The cycling program for the amplification of 321 bp of ITS-2 included an initial denaturation at 95°C for 2 min, followed by 30 cycles of denaturation at 95°C for 1 min, annealing at 55°C for 1 min, extension at 72°C for 1 min, and a final extension at 72°C for 7 min. The initial denaturation for the *nad*4 gene was 94°C for 5 min, followed by 30 cycles of denaturation at 94°C for 30 s, annealing at 55°C for 30 s, extension at 72°C for 1 min, and a final extension of 72°C for 5 min. A 1.5% agarose gel (w/v) was prepared using 1 x TAE buffer and 5 μl of ethidium bromide (0.5 μg/μl) was added to 50 ml of agarose gel based on the manufacturer’s guidelines. The PCR amplicons (5 μl) were analyzed on the gel and a 5-μl 100 bp sized DNA marker was used. The bands of all PCR amplicons were visualized and compared with the gene marker in a UV light chamber.

**Table 1:** The sequences of the primers used, along with their characteristics on sequence length (bp), Tm value (°C), GC content (%), and concentration (pmol/μl) of the working volume of the primer

Primer	Sequence (5′–3′)	Length (bp)	Tm value (°C)	GC (%)	Concentration (pmol/μl)
NC1-F	ACGTCTGGTTCAGGGTTGTT	20	52	50	10
NC1-R	TTAGTTTCTTTTCCTCCGCT	20	48	40	10
Primer1-F	GGATTTGGTCAGCAAATTGAA	21	49	38	10
Primer2-R	GCCTGCAAATGAATTAACA	19	45	37	10

Positive PCR products were purified using the FavorPrep^TM^ PCR Clean-Up Mini Kit (iNtRON Biotechnology, Korea) according to the manufacturer’s guidelines. All centrifugation was performed at maximum speed (14,000 rpm) in a microcentrifuge (Type-ScanSpeed Mini; Article-7.601.314.101). Finally, 40 μl of elution buffer was used to elute the pure DNA. The PCR-positive DNA was then sequenced directly ((BigDye^TM^ Terminator v.3.1 cycle sequencing kit, Applied Biosystems, USA) on an automated sequencer (ABI3730XL, Applied Biosystems) using the forward and reverse primers in separate reactions.

### Data analysis

The ITS-2 and *nad*4 gene sequences were analyzed using the NCBI BLAST program (http://www.ncbi.nlm.nih.gov/). After editing the sequences in DNA Baser program (version 3.1) and deleting unresolved nucleotides, multiple alignments were performed using Clustal W in MEGA 7.0 (Tamura et al. 2004). Genetic distances, nucleotide diversity indices, and bootstrapped confidence limits were estimated using the neighbor-joining method (Saitou and Nei 1987).

The nucleotide diversity indices were used to estimate the genetic diversity of the population. Confidence limits were assessed using the bootstrap procedure (Felsenstein 1985) with 1200 replicates for the NJ method. Codon positions included were 1st, 2nd, 3rd, and noncoding in the case of the *nad*4 gene. All positions containing gaps and missing data were eliminated. A sequence of *H. contortus* was retrieved from GenBank (Accession No. KJ724453) for comparison, and a sequence of *H. placei* (Accession No. AF070820) was selected as an outgroup taxon. A 50% consensus cut-off value for the tree was implemented. The accession numbers for the ITS-2 sequences for surrounding countries of Bangladesh were retrieved from the GenBank database and used for constructing the phylogenetic tree and conducting pairwise distance analysis (Table 2). The diversity indices were calculated using the program DnaSP5.1 (Librado and Rozas 2009) to evaluate degree of gene flow among populations. Tajima’s D (Tajima 1989) was also calculated to test neutrality using the same program (DnaSP 5.1).

**Table 2:** The accession numbers of the ITS-2 sequences derived from the Genbank database along with the nucleotide diversity (*μ*) within each population of *H. contortus* for China, India, Pakistan, Thailand, and this study

Country	Accession number	Sequences no.	Nucleotide diversity (*π*)
Bangladesh	KU558755–KU558759	5	0.009524
China	KC415118–KC415120, KC415125, KC415129	5	0.012121
India	KJ857556–KJ857560	5	0.452966
Pakistan	KJ724250, KJ724320, KJ724322–KJ724323, KJ920742	5	0.0000
Thailand	KP101369–KP101371, KP101378–KP101379	5	0.013853

## Results

### Sequence analyses and genetic variation

A fragment of 321 bp of the ITS-2 region of the nuclear rDNA and 800 bp of the *nad*4 gene was amplified to identify *H. contortus*. The length of the PCR amplicons of *H. contortus* was 321 bp, consisting of 231 bp of the ITS-2 and 90 bp of the flanking regions (20 bases from the 3′ end of the 5.8S and 70 bases from the 5′ end of the 28S gene). The ITS-2 sequences were analyzed, and 231-bp lengths were obtained after editing each sequence. All the sequences were aligned. The sequence identities ranged from 98.5 to 100% when compared with the reference (KM586651) sequences of *H. contortus*. These sequences were also compared with two reference ITS-2 sequences for *H. placei* (accession numbers AJ577466 and AM410068), and 96% nucleotide identities were found for each. A 97% nucleotide identity was observed when compared with the ITS-2 sequence for *H. bedfordi* (accession number KP688065).

The alignment of the sequences from this study (KU558755-KU558759) with the reference sequence (accession number KM586651) revealed four substitutions at positions 30, 41, 42, and 216 of the nucleotides. There was one transition and three transversions among the substitutions. The transition was found between cytosine (C) and thymine (T) of the pyrimidine at position 42 of the nucleotides. The transversions were found at positions 30 (C/A), 41 (G/C), and 216 (T/A) of the nucleotides. The *nad*4 gene sequences were aligned with the reference sequence (accession number KJ724453). The sequence identities ranged from 98.5 to 100% nucleotide identities. The genetic variations (substitutions) in the 45 aligned *nad*4 gene sequences with the reference sequence were found at positions 87, 106, 180, 183, 192, 193, 201, 243, 465, 474, 555, 603, 627, 645, and 657 of the nucleotides, and all variations were transitions. Ten transitions were found between purines, and five transitions were found between pyrimidines. The nucleotide diversity (*π*) among the 10 ITS-2 and 45 *nad*4 sequences of *H. contortus* from Bangladesh was 0.0095 and 0.003946, respectively (Table 3). A total of 55 nucleotide sequences were submitted to the European Nucleotide Archive (ENA) and the DNA Data Bank of Japan under the accession numbers KY031955-KY031964 and KY041808-KY041842 for *nad*4 and KU558755-KU558759, KU870651-KU870653, and KU640184-KU640185 for ITS-2.

**Table 3:** The nucleotide diversity (*π*) within each population was calculated using the *nad*4 gene retrieved from the GenBank database for four countries (China, Malaysia, Yemen, and the USA), and this study

Country	Accession number	Sequences no.	Nucleotide diversity (*π*)
Bangladesh	KY041808–KY041842, KY031955–KY031964	45	0.00394
China	KC429944–KC430085	142	0.02745
Malaysia	HQ660255–HQ660308	54	0.03545
Yemen	HQ660309–HQ660367	59	0.03462
USA	AF070736–AF070785	46	0.02512

### Phylogenetic analysis

**Fig. 1:**
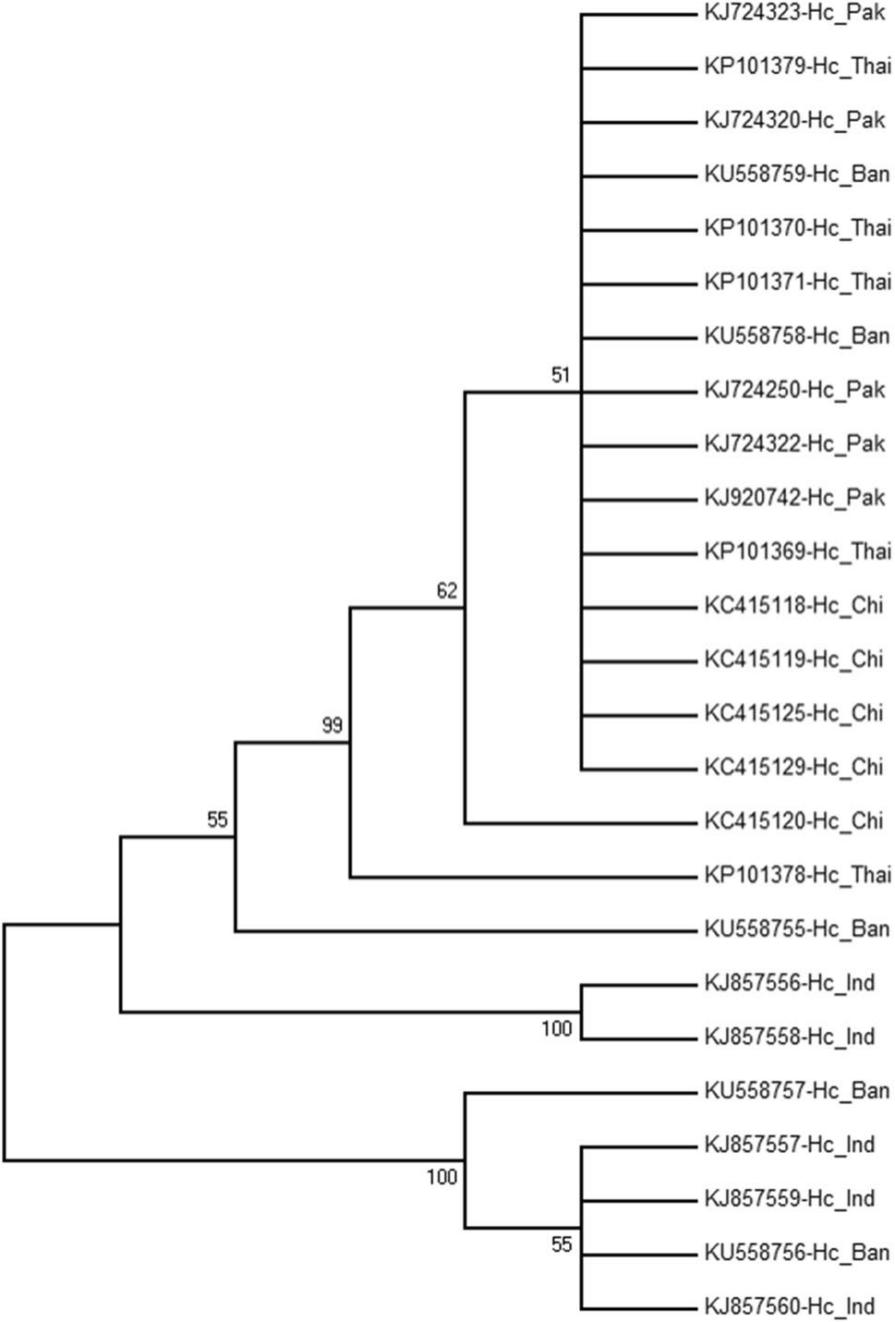
A phylogenetic tree was constructed using partial nucleotide sequences of the 25 ITS-2 gene of *H. contortus*. Five sequences were used for each of the countries of Bangladesh (Ban), China (Chi), India (Ind), Pakistan (Pak), and Thailand (Thai). The sequences were retrieved from the GenBank database, and evolutionary analyses were conducted in MEGA7 (Kumar et al. 2016)

**Fig. 2:**
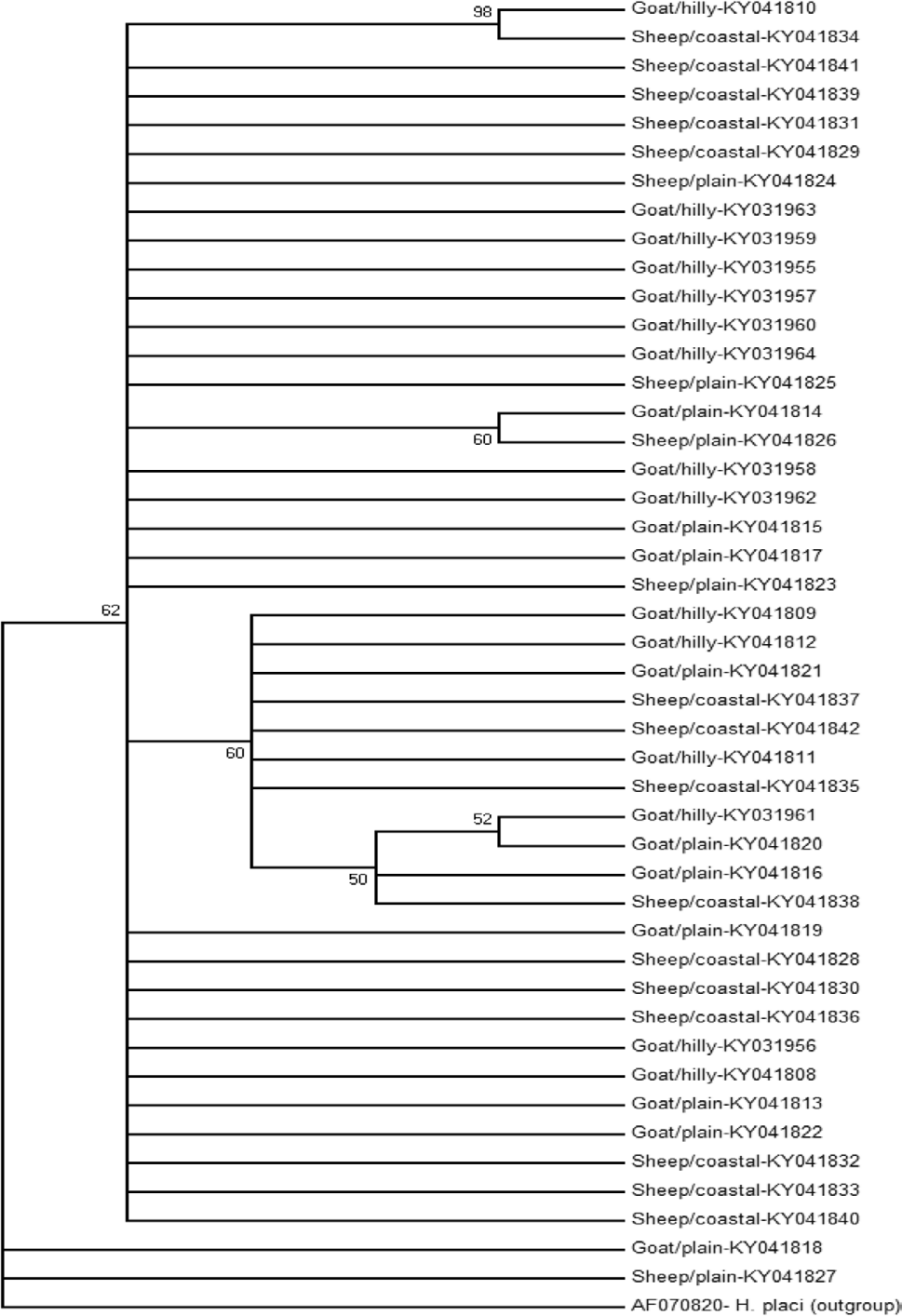
The phylogenetic tree displays the relationship among the 45 *nad*4 sequences of *Haemonchus contortus* from three geographical regions in Bangladesh. Each terminal branch represents a single sequence, which is labeled according to its geographical origin and host of the worm from which it was derived. *Haemonchus placei* (accession number AF070820) was used as the outgroup

The phylogenetic tree was constructed using 25 sequences of ITS-2 for *H. contortus* using NJ method (Fig. 1). The 5 sequences used randomly for each country (Bangladesh, China, India, Pakistan, and Thailand). One cluster obtained all sequences of Pakistan and China with moderate nodal support (62%) while it was observed that Thailand was incorporated with Pakistan and China with strong nodal support (99%). Another cluster contained 2 sequences of Bangladesh and 3 sequences from India with strong nodal support (100%). The phylogenetic tree was constructed using 45 sequences of *nad*4 gene using NJ method (Fig. 2) with the *nad*4 sequence of *H. placei* (accession no. AF070820) used as the outgroup. The tree reveals information about the inferred evolutionary relationships and has a sum of branch length of 0.02497802. The taxa representing various regions are randomly distributed with poor support (< 50%) for some nodes. The results show low genetic variation among the *H. contortus* populations in Bangladesh and there are no obvious boundaries among individuals in the trees, with only some individuals having moderate nodal support (60%).

### Evolutionary divergence between sequences

The number of base substitutions per site from between sequences is shown in Table 4. The pairwise distances among the 10 sequences of *H. contortus* ranged from 0.005 to 1.477. Average evolutionary divergence over all sequence pairs was 0.761, obtained by bootstrap procedure (1000 replicates) described by Tamura et al. (2004). All species showed low genetic divergence. Diversity and neutrality indices for different populations of *H. contortus* from Bangladesh were also calculated from *nad*4 nucleotide data sets (Table 5).

**Table 4:** Pairwise distances (%) of the partial sequences of the ITS-2 gene for *H. contortus* isolated from small ruminants in Bangladesh. The ITS-2 gene of *H. contortus* isolated from Bangladesh was compared with those from India (KJ857557) and Pakistan (KJ724250), and the sequences were retrieved from the GenBank. Evolutionary analyses were conducted using MEGA7 (Kumar et al., 2016)

	1	2	3	4	5	6	7	8	9	10	11	12
1. KU558756; Ban-G	-											
2. KU558757; Ban-G	0.010	-										
3. KU558758; Ban-G	0.010	0.000	-									
4. KU558759; Ban-G	0.010	0.000	0.000	-								
5. KU870652; Ban-G	1.384	1.385	1.385	1.385	-							
6. KU558755; Ban-S	0.005	0.005	0.005	0.005	1.355	-						
7. KU870651; Ban-S	1.413	1.413	1.413	1.413	0.026	1.382	-					
8. KU640184; Ban-S	1.354	1.355	1.355	1.355	0.016	1.325	0.026	-				
9. KU640185; Ban-S	1.449	1.449	1.449	1.449	0.021	1.416	0.010	0.032	-			
10. KU646834; Ban-S	1.413	1.413	1.413	1.413	0.026	1.382	0.010	0.032	0.010	-		
11. KJ857557; Ind-G	1.477	1.477	1.477	1.477	0.048	1.442	0.032	0.037	0.026	0.032	-	
12. KJ724250; Pak-S	1.477	1.477	1.477	1.477	0.048	1.442	0.032	0.037	0.026	0.032	0.000	-

*Ban*, Bangladesh; *Ind*, India; *Pak*, Pakistan; *G*, goat; *S*, sheep

**Table 5:** The diversity and neutrality indices for different populations of *Haemonchus contortus* from different geographical locations in Bangladesh were calculated from the *nad*4 nucleotide data sets

Location	*nad*4 observed	nucleotide diversity (*π*)	Tajima’s *D*
Plain	15	0.004363	−0.537933
Hilly	15	0.004102	−0.990972
Coastal	15	0.003684	−0.794421

## Discussion

*Haemonchus contortus* was identified by amplifying ITS-2 and partial *nad*4 sequences of these parasites that were collected from small ruminants raised in different geographical regions of Bangladesh. The sequence analysis showed variations in ITS-2 among the worms from all locations, with sequence similarities ranging from 98.5 to 100% when compared with the reference sequence with accession number KM586651. Four substitutions were found at the 30th, 41st, 42nd, and 216th nucleotide positions. The ITS-2 isolated from *H. contortus* in the veterinary field showed more variation than previously reported in studies by Von Samson-Himmelstjerna et al. (2002), Rahman et al. (2007), Cerutti et al. (2010), and Akkari et al. (2013). In the observation of Yin et al. (2013) in China, there were 6 substitutions at the nucleotide positions 10, 18, 21, 22, 123, and 196 from the 18 aligned sequences. These substitutions included 2 transitions (T/C) and 4 transversions (one A/C, one G/C, and two A/T).

We also found more transversions than transitions. This might be due to a natural attraction (A/T; G/C) towards a desired nucleotide during DNA synthesis. These variations were in agreement with an earlier published report on the genetic variation of *H. contortus* by Gharamah et al. (2012). They observed three nucleotide substitutions at positions 6, 108, and 181 from 80 aligned sequences in Perak sheep and goats in Malaysia. These nucleotide variations were not in the same nucleotide positions, while Stevenson et al. (1995) and Cerutti et al. (2010) found transitions between purines (G/A) at positions 22, 202, and 216. The alignment between ITS-2 sequences of *H. contortus* and *H. placi* indicated a high degree of nucleotide diversity and showed 96% nucleotide identities. A 97% nucleotide identity was observed when compared with the ITS-2 sequence for *H. bedfordi* (accession no. KP688065). Low genetic differentiations were also found in the case of the *nad*4 gene.

For *nad*4, the nucleotide diversities within each of the *H. contortus* populations in three regions ranged from 0.003684 to 0.004363. The average nucleotide diversity of 0.004049 for the *nad*4 gene in *H. contortus* populations in three regions was slightly lower than the previously published data for this mitochondrial gene in countries like Malaysia (0.032–0.044) and Yemen (0.021–0.036), according to the observation of Gharamah et al. (2012).

The phylogenetic tree was constructed to observe the evolutionary relationships using ITS-2 among surrounding countries of Bangladesh. The tree revealed continuous variation in genetic distance among nucleotide sequences, with a random distribution of sequences representing various locations in these countries. There was an exception in some isolates from China and Thailand, which showed a high degree of genetic diversity. This was in agreement with the results obtained by Gharamah et al. (2012), who found distinct relations among populations of *H. contortus* in Malaysia and Yemen. The phylogenetic analysis of *H. contortus* was not clear in terms of grouping based on host species, whereas Yin et al. (2013) observed genetic variability within and among populations of *H. contortus* in China. The phylogenetic tree was also constructed using 45 *nad*4 sequences to observe the evolutionary relationships, which revealed little genetic deviation among the populations of *H. contortus* in Bangladesh. This was in concurrence with the results obtained by Yin et al. (2013) in China. In general, the results showed low genetic differentiation and high gene flow among the populations of *H. contortus* in Bangladesh.

The pairwise distances among the 12 sequences of *H. contortus* ranged from 0.005 to 1.477, with an average evolutionary divergence of 0.761. According to Gharamah et al. (2012) in Malaysia, the pairwise distances among the eight populations of *H. contortus* ranged from 0.004 to 0.006, which showed low genetic divergence and suggested that this gene was not suitable for population studies. The limitation of this study was the limited availability of high-quality samples for selection in sequencing. Furthermore, we preferred samples with high-quality DNA that were free of contaminants and had a high yield for sequencing.

## Conclusion

We have shown that the low nucleotide diversity observed in Bangladesh was compared to surrounding countries. The successful amplification of 321 bp of ITS-2 from the nuclear rDNA and *nad*4 gene of *H. contortus* was conducted to facilitate molecular detection of the parasite. The results showed low genetic variation among the *H. contortus* population in Bangladesh and have crucial implications for studying molecular epidemiology and developing control strategies against haemonchosis.

## Data Availability

Representative nucleotide sequences obtained in this study were submitted to the European Nucleotide Archive (ENA) and the DNA Data Bank of Japan under the accession numbers KY031955-KY031964 and KY041808-KY041842 for *nad*4 and KU558755-KU558759, KU870651-KU870653, and KU640184-KU640185 for ITS-2.
